# Characterization of the Microenvironment in Positive and Negative Sentinel Lymph Nodes from Melanoma Patients

**DOI:** 10.1371/journal.pone.0133363

**Published:** 2015-07-28

**Authors:** Meriem Messaoudene, Aurélie Périer, Giulia Fregni, Emmanuelle Neves, Laurence Zitvogel, Isabelle Cremer, Johan Chanal, Xavier Sastre-Garau, Lydia Deschamps, Eduardo Marinho, Frederique Larousserie, Eve Maubec, Marie-Françoise Avril, Anne Caignard

**Affiliations:** 1 INSERMU1160, Institut Universitaire d’Hématologie, Hôpital Saint Louis, 1 Avenue Claude Vellefaux, 75010, Paris, France; 2 U1015 INSERM-CIC, Institut Gustave Roussy, Villejuif, France; 3 Centre de Recherche des Cordeliers, 15, rue de l'école de Médecine, 75006, Paris, France; 4 APHP, Department of Dermatology, Hospital Cochin, University Paris Descartes, Paris, France; 5 Biopathology Department, Institut Curie, Rue d’Ulm, Paris, France; 6 APHP, Department of Dermatology and Department of Pathology, Hospital Bichat, University Paris Diderot, Hospital Bichat, 75018, Paris, France; 7 APHP, Department of Pathology, Hospital Cochin, University Paris Descartes, Paris, France; University of Palermo, ITALY

## Abstract

Melanomas are aggressive skin tumors characterized by high metastatic potential. Our previous results indicate that Natural Killer (NK) cells may control growth of melanoma. The main defect of blood NK cells was a decreased expression of activating NCR1/NKp46 receptor and a positive correlation of NKp46 expression with disease outcome in stage IV melanoma patients was found. In addition, in stage III melanoma patients, we identified a new subset of mature NK cells in macro-metastatic Lymph nodes (LN). In the present studies, we evaluated the numbers of NK cells infiltrating primary cutaneous melanoma and analyzed immune cell subsets in a series of sentinel lymph nodes (SLN). First, we show that NKp46^+^ NK cells infiltrate primary cutaneous melanoma. Their numbers were related to age of patients and not to Breslow thickness. Then, a series of patients with tumor-negative or -positive sentinel lymph nodes matched for Breslow thickness of the cutaneous melanoma was constituted. We investigated the distribution of macrophages (CD68), endothelial cells, NK cells, granzyme B positive (GrzB^+^) cells and CD8^+ ^ T cells in the SLN. Negative SLN (SLN^-^) were characterized by frequent adipose involution and follicular hyperplasia compared to positive SLN (SLN^+^). High densities of macrophages and endothelial cells (CD34), prominent in SLN^+^, infiltrate SLN and may reflect a tumor favorable microenvironment. Few but similar numbers of NK and GrzB^+^ cells were found in SLN^-^ and SLN^+^: NK cells and GrzB^+^ cells were not correlated. Numerous CD8^+^ T cells infiltrated SLN with a trend for higher numbers in SLN^-^. Moreover, CD8^+^ T cells and GrzB^+^ cells correlated in SLN^-^ not in SLN^+^. We also observed that the numbers of CD8^+ ^ T cells negatively correlated with endothelial cells in SLN^-^. The numbers of NK, GrzB^+^ or CD8^+^ T cells had no significant impact on overall survival. However, we found that the 5 year-relapse rate was higher in SLN with higher numbers of NK cells.

## Introduction

Melanoma is an aggressive skin tumor characterized by high metastatic potential. During the disease progression, the sentinel lymph node (SLN) represents the first metastatic site, since it is the first regional lymph node that receives lymph fluid from the primary melanoma. SLN biopsy procedure is an important prognostic and staging tool in melanoma patients with tumors more than 1 mm in thickness [1]. However, besides radical lymph node dissection, there is no established therapeutic protocol for adjuvant treatment of micrometastatic SLN (SLN^+^) patients. Beyond its prognostic value, SLN represents an important immune system headquarter, especially for the T-cell priming and differentiation and maturation of NK cells. Therefore, the analysis of the immunological parameters (localization of immune cells and interactions with other cell types) and their prognostic impact are crucial to consider, for setting up future designs of adjuvant immunotherapy. Several studies have investigated T cell, dendritic cell and macrophage distribution and reactivity in SLN^+^ from melanoma, breast cancer and gastric cancer patients [2–7]. In contrast, rare studies analyzed NK cells, mainly because, until recently, there was no recognized single marker staining to detect effectively these cells *in situ*.

NK cells, defined as CD3^-^CD56^+^ lymphocytes, are central components of the innate immunity mediating spontaneous “natural” cytotoxicity toward tumor and virus-infected cells. They display preformed cytoplasmic granules that contain cytotoxic proteins such as granzymes. They also display immunomodulatory functions through secretion of chemokines and cytokines, like IFNγas well as TNFα and GM-CSF, controlling the adaptive immune response [8].

We have obtained previous results indicating a role of NK cells in the immune response in melanoma patients. We have shown that blood NK cells from stage IV metastatic melanoma patients exhibit a decreased NKp46 expression and altered functional abilities [9]. We described progressive alterations of blood NK cells in melanoma patients according to clinical stages (stage I to IV) with a significant decrease of NKp46 expression on blood NK cells from stage IV metastatic melanoma patients and an alteration of their functional abilities [10]. In addition, we found that a decreased NKp46 expression correlates with shorter duration of stage IV. Our results also showed that NK cells infiltrate primary melanoma [10].

Furthermore, we have recently identified a new subset of NK cells infiltrating macro-metastatic lymph nodes from melanoma patients. We found that terminally mature CD56^bright^CD16^+^NCR^+^ NK cells infiltrated metastatic LN and activated by cytokines *ex vivo*, efficiently lysed metastatic lymph node derived melanoma cells [11].

These results led us to quantitate *in situ* the NK cells infiltrating SLNs and to compare their numbers and distribution in SLN^+^ and SLN^-^ and in primary cutaneous melanomas. We used anti-NKp46 mAb (Natural Cytotoxicity Receptor, NCR1), a marker of the NK cell lineage, to stain NK cells *in situ*. This mAb was previously used to evaluate the numbers of NK cells infiltrating tissues and various tumors. NKp46^+^ cells were found in splenic red pulp, lymph nodes, lungs, and gut lamina propria from healthy donors [12], in lung cancers and GIST [13,14]. Moreover, others cytotoxic immune cells (CD8^+^ T cells and Granzyme B^+^ cells) were enumerated and the density and distribution of macrophages (CD68^+^ cells) and endothelial cells (CD34^+^ cells) were also evaluated in the SLNs. The results on the characterization of immune cell subsets in SLNs and primary cutaneous melanoma were discussed taking into account the clinical evolution of the patients.

## Material and Methods

### Patients and samples

The analyzed series consisted in 39 primary cutaneous melanomas (Table 1) and in SLN samples, either micro-metastatic (SLN^+^) or normal (without tumor cells) SLN (SLN^-^) from melanoma patients (Table 2). All SLNs were obtained from patients with cutaneous melanoma of >1 mm in thickness who had undergone SLN dissection between April 2006 and June 2009 in two APHP hospitals (Bichat and Cochin hospitals) (Table 2). In the present studies, melanoma patients with at least 5-years follow-up were included. Among the 86 patients of the SLN series, 63 had SLN^-^ and 23 patients had a SLN^+^ reflecting the proportions of positive and negative sentinel LN in patients with thick cutaneous melanoma. SLN^-^ and SLN^+^ patients were paired according to Breslow thickness and the proximity of surgery dates. They were not paired according to sex, tumor ulceration or Clark index neither according to localization on the body (Table 2). Patients did not receive any anticancer treatment prior to SLN surgery. SLN procedure was performed as part of the initial treatment of the primary melanoma. The series contains 41 females and 45 males, SLN^+^ were present in 14 males and 9 females and SLN^-^ were present in 31 males and 32 females. The study protocol (Program IMMUMELA) and patient consent procedure were approved by an ethic committee (Comité de Protection des Personnes Ile de France CPP: 2834) and the declaration of Helsinki protocols were followed. The study was explained to the patient who also received a written information sheet; thereafter the patient signed an informed consent.

**Table 1:** Characteristics of the cutaneous melanoma and the clinical evolution of patients included in the studies of primary melanomas. SSM: Superficial Spreading Melanoma; NM: Nodular Melanoma; ALM: Acral Lentiginous Melanoma.

	All patients (n = 39)
**Age**	
Mean	64.02 (21–91)
Median	68
**Sex**	
Male	19
Female	20
**Tumor thickness (mm)**	
Mean	4,62 (0.5–27)
Median	3.3
0.5–2.0	11
2.0–4.0	14
> 4.0	14
**Histology**	
SSM	19
NM	10
ALM	4
Spitzoïd	1
mucosal	2
unknown	3
**Ulceration**	
Present	23
Absent	11
Unknown	5
**Progression in 5 years**	
No	19
All stage	20
Stage III	9
Stage IV	11
**5-years Survival (%)**	43

**Table 2:** Characteristics of the cutaneous tumors of the patients included in the SLN cohort and their clinical evolution over 5 years follow-up. SSM: Superficial Spreading Melanoma; NM: Nodular Melanoma; ALM: Acral Lentiginous Melanoma.

** **	**All patients (n = 86)**	**SLN– (n = 63)**	**SLN+ (n = 23)**
**Age**			
Mean	54.9	54.9	53.6
Median	55.7	56.3	41
**Sex**			
Male	45	31	14
Female	41	32	9
**Tumor thickness (mm)**			
Mean	2.9	2.69	3.41
Median	2.1	2.1	2.4
1.0–2.0	42	34	9
2.0–4.0	24	17	7
> 4.0	19	12	7
**Tumor site**			
Head and neck	12	9	3
Trunk	25	18	7
Extremities	48	35	13
**Histology**			
SSM	43	33	10
NM	34	26	8
ALM	6	3	3
Unknown	3	1	2
**Ulceration**			
Present	34	24	10
Absent	49	37	12
Unknown	3	2	1
	**All patients (n = 86)**	**SLN**–** (n = 63)**	**SLN+ (n = 23)**
**Progression in 5 years**			
No	59	45	12
All stage	27	18	11
Stage III	13	7	7
Stage IV	14	11	4
**5-years Survival (%)**	77	88	69

### Immunohistochemical detection of immune cell types

Serial 3-μm thick sections from formalin-fixed paraffin-embedded samples were mounted on poly-l-lysine–coated slides. Serial slides were stained with monoclonal antibodies against NKp46 (R&D systems), Granzyme B (GrzB^+^) (AbCAM), CD8 (SpringBioscience), CD34 (Immunotech), and CD68 (Dako). For antigen retrieval, slides were deparaffinized with xylene, and rehydrated through graded alcohols to water ratio rinces. The slides were then incubated for 30 min in a 98°C water bath with retrieval buffers consisted of Tris-EDTA buffer (10 mmol/L Tris and 1 mmol/L EDTA) pH 9 for NKp46 and GrzB or pH 8 for CD8 staining. Slides were washed for 30 min in a 98°C water bath. Endogenous peroxidase activity was inhibited with 3% hydrogen peroxidase (Dako) for 10 min and nonspecific protein were blocked for 15 min (Protein Block; Dako). The primary antibody was incubated for 1 h, followed by the secondary antibody, polymer-peroxidase for mouse (NKp46 and GrzB) or rabbit (CD8) monoclonal antibody (EnVision System; Dako) incubated for 45 min. Peroxidases were revealed with 3-amino-9-ethylcarbazole substrate (AEC; Vector Laboratories). Finally the slides were mounted using Glycergel (Dako). CD68 (macrophages) and CD34 (endothelial cells) staining was carried out on fully automated system following manufacturers' recommendations in the pathology department of Bichat hospital.

### Method for cell quantification

Macrophages (CD68^+^) and endothelial cells (CD34^+^) were too abundant in SLN to be counted as single cells, the staining sections were scored on a scale representing the estimated proportion of positive cells noted (+: moderate, ++: high and +++: intense density).

For NKp46^+^ NK cells and GrzB^+^ cells, stained sections were scanned using NanoZoomer (Hamamatsu Photonics): sections were scanned at low magnification. NKp46^+^ and GrzB^+^ cells were then counted using NDP software in SLN^-^, in the center of the tumor (intratumoral) and in the peritumoral areas for SLN^+^ and primary invasive cutaneous melanomas in at least 15 fields. Areas with the highest density of positive cells (hot spots) were counted at 400x magnification. Counting was performed by an investigator, blinded to the clinical information. Numbers of CD8^+^ T cells were counted automatically on the stained sections (Hamamatsu Photonics) using CaloPix software.

### Statistical analysis

Statistical tests and graphics were generated by Prism version 5 (GraphPad Software Inc). Nonparametric unpaired Mann-Whitney (M-W) test was used to compare SLN^-^ and SLN^+^. Wilcoxon matched paired test was used to compare intratumoral and peritumoral areas in the SLN^+^ and in the primary cutaneous melanomas. Non-parametric Kruskal-Wallis (K-W) test was used to compare the 3 different groups (p values noted as * p≤0.05, ** p<0.01 and *** p<0.0001). The survival curves were plotted according to the Kaplan-Meier method and compared using the Log-rank test.

Correlations between cytotoxic immune cells (NKp46^+^ cells, GrzB^+^ cells and CD8^+^ cells) infiltrating the SLN^-^ and the SLN^+^ samples were assessed. The correlations of numbers of NKp46^+^ cells with the Breslow and the age of melanoma patients were analyzed by nonparametric Spearman test.

## Results

### NK cells infiltrate primary cutaneous melanoma tumors: no impact of Breslow on NK cell numbers

**Fig 1:**
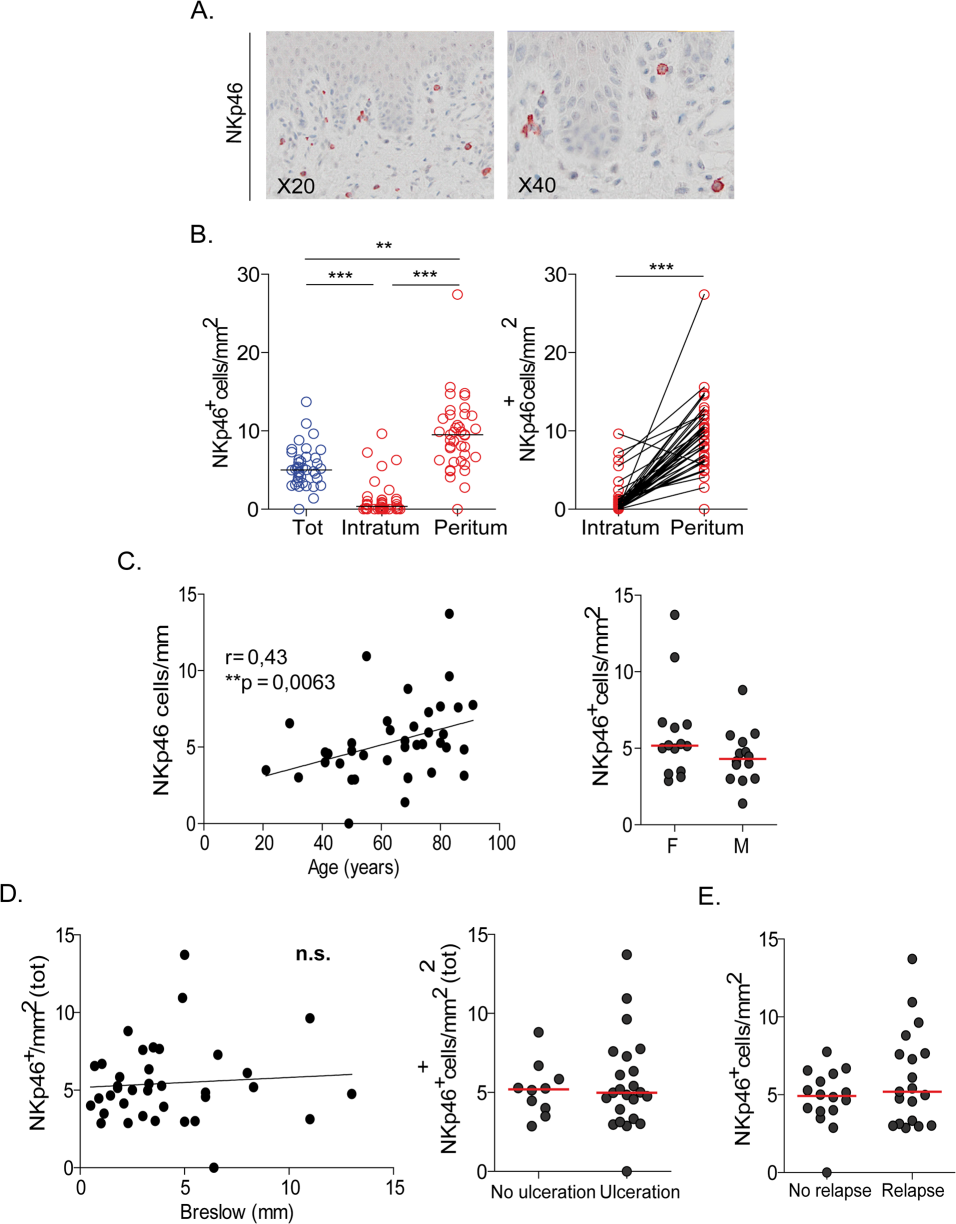
Immunohistological staining and counting of NKp46^+^ NK cells in primary cutaneous melanomas. (A) Representative staining of NK cells with anti-NKp46 mAb. (B) Enumeration of NK cell numbers infiltrating cutaneous melanoma (left panel) and paired analysis of intra and peritumoral NK cells (right panel). (C) Correlation of NK cell numbers and age (left panel) or gender (right panel) of the patients. (D) Correlations of NK cell numbers with Breslow thickness (left panel), or with presence of tumor ulceration (right panel). (E) Correlation of NK cell numbers and 5 years-relapse rates.

Table 1 summarizes the characteristics of the 39 cutaneous primary melanoma samples analyzed. Numerous NK cells infiltrated primary cutaneous melanomas and corresponded to large granular cells displaying bright red staining with anti-NKp46 mAb (Fig 1A). A mean value of 5.3 NK cells/mm^2^ was enumerated in primary cutaneous melanomas (Fig 1B). The density of NK cells was higher in peritumoral regions (9.5 NK/mm^2^) than in tumor areas that contained no (4/39 tumors) or rare NK cells (1.24 NK/mm^2^) (Fig 1B). In this series of primary cutaneous melanomas, we observed a positive correlation between numbers of NKp46^+^ cells and the age of the patients (Fig 1C, left panel) but not according to the gender (Fig 1C, right panel). In addition, as depicted in Fig 1D, we found no correlation of NK cell numbers and the tumor characteristics (ie. Breslow thickness or the presence of ulceration). Moreover, the numbers of NKp46^+^ cells infiltrating the primary melanomas did not correlate with the occurrence of 5-years relapse (Fig 1E).

### Characteristics of primary cutaneous melanoma of patients included in the SLN cohorts

Patients treated by SLN procedure with a long follow-up superior to 5 years were included. The characteristics of the cutaneous melanoma of these patients are summarized in Table 2. According to our selection criteria, SLN^+^ and SLN^-^ groups displayed comparable proportions of patients with intermediate or thick melanomas. The mean Breslow thickness was 2.7 mm in SLN^-^ and 3.4 mm in SLN^+^. The Kaplan-Meier survival curves indicate that higher Breslow (> 3.49mm) correlated with a decreased overall survival (OS) and progression free survival (PFS) (S1A Fig). Furthermore, alike the series of primary cutaneous melanomas (Fig 1), high Breslow was associated with the age of the patients and ulcerated tumors (S1B and S1C Fig) and was also correlated with increased 5 years relapses (S1D Fig). The Kaplan-Meier survival curves show that the SLN status had no significant impact on OS or PFS (S1E Fig). The localizations of the primary melanoma (extremities, trunk, head and neck) were comparable in the SLN^-^ and SLN^+^ groups. Ulceration of the primary melanoma was present in 48% of the SLN^+^ group and in 41% of the SLN^-^ group (Table 2). Among SLN^+^, 12/23 displayed rare isolated tumor cells while 8/23 contained large clusters of tumors cells.

### High densities of macrophages and endothelial cells infiltrate SLN

Among the SLN samples, a series of 61 SLN samples (41 SLN^-^ and 20 SLN^+^) was analyzed for CD34 and CD68 staining. The morphological analysis of these SLNs on HES (haematoxylin-eosin-saffron) stained slides outlined distinct characteristics of SLN^-^ compared to SLN^+^. Numerous SLN^-^, 33 out of 41 (80%) showed adipose involution and 36/41 (87%) displayed follicular hyperplasia whereas 13/20 (65%) SLN^+^ had adipose involution and 12/20 (60%) displayed follicular hyperplasia, suggesting more frequent signs of immune reaction in SLN^-^ (Table 3).

**Table 3:** Histological characteristics of SLN (HES staining) and distribution and staining density of macrophages (CD68) and endothelial cells (CD34) in SLN^-^ and SLN^+^ in the different areas (inter-, intra-follicular and subcapsular) of the SLN.

	SLN- (n = 41)	SLN+ (n = 20)
**Tumor cells**	NO	12/20 (tumor cell clusters) = 60%
**adipose involution**	33/41 (80%)	13/20 (65%)
**Follicular hyperplasia**	36/41 (87%)	12/20 (60%)
**CD68+ cells**	**Inter-follicular**	**Inter-follicular**
** **	(+++) 20/41	(+++) 12/20
** **	(++) 19/41	(++) 6/20
** **	(+) 3/41	
** **	**Intra-follicular**	**Intra-follicular**
** **	(+++) 3/41	(+++) 2/20
** **	(++) 12/41	(++) 9/20
** **	(+) 20/41	(+)7/20
** **	**Subcapsular**	**Subcapsular**
** **	(+++) 13/41	(+++) 14/20
** **	(++) 13/41	(++) 2/20
** **	(+) 8/41	
** **		**Intra-tumor**
** **		(++) 3/6
** **		(+) 3/6
**CD34+ cells**	**Inter-follicular**	**Inter-follicular**
** **	(+++) 22/41	(+++) 17/20
** **	(++) 7/41	(++) 3/20
** **	(+) 11/41	
** **		**Intra-tumor**
** **		(++) 3/6
** **		(+) 3/6

Staining intensity was evaluated as score: (+) moderate, (++) high) and (+++) intense.

**Fig 2:**
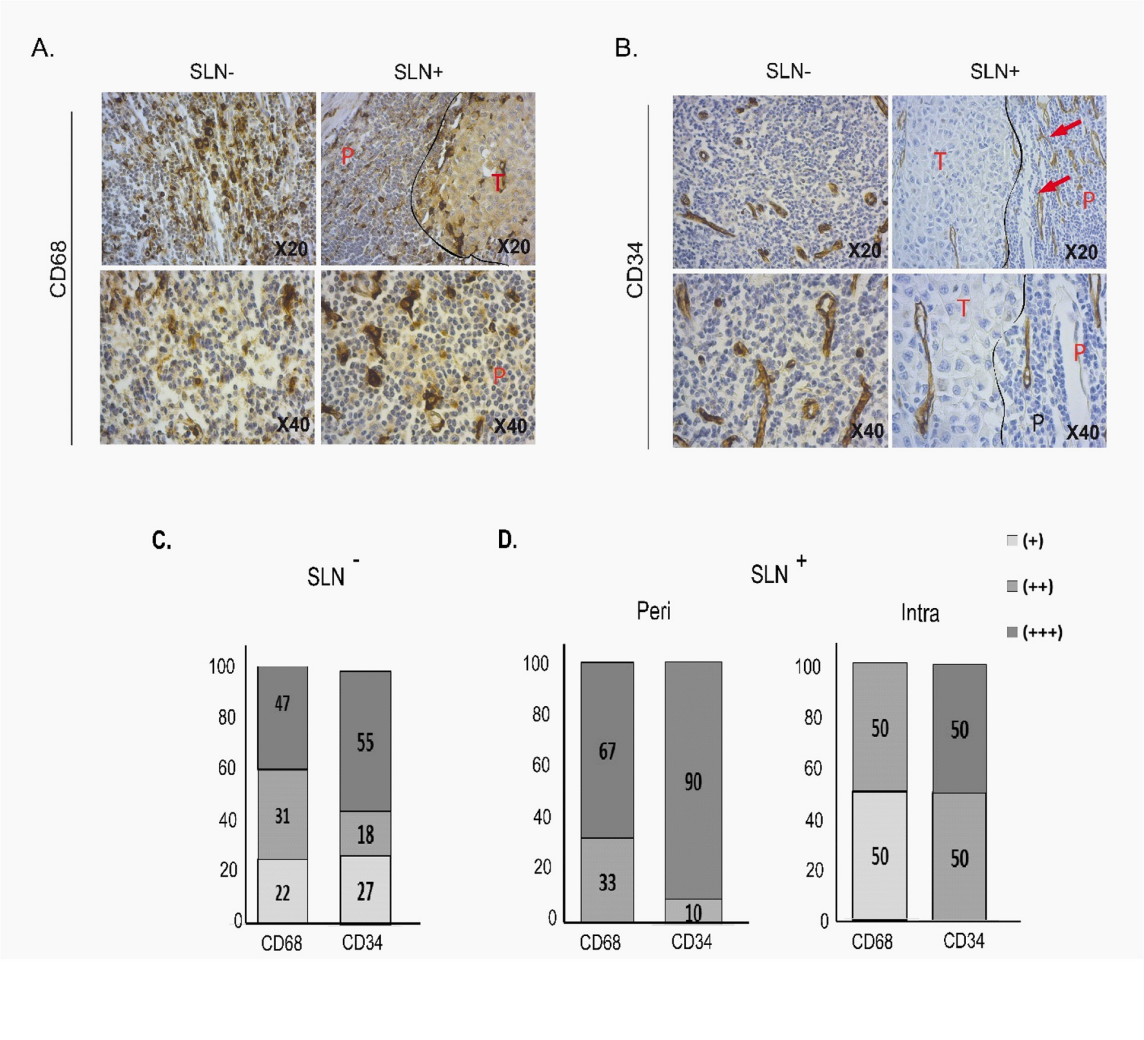
Immunohistological staining and density evaluation of macrophages (CD68) and endothelial cells (CD34) in SLN. Due to the high numbers of labelled cells, staining was evaluated as score: (+) moderate, (++): high, (+++): intense staining. Representative staining (X20 and X40) with (A) anti-CD68 and (B) anti-CD34 in SLN (P: Peritumor area; T: Tumor area). Percentages of CD68 and CD34 density staining in SLN^-^ (C) and SLN^+^ (D) in peritumor (Peri) (left panel) and intratumor (Intra) areas (right panel).

The SLN are characterized by high densities of macrophages (CD68^+^) and endothelial cells (CD34^+^) and differences in the distribution of these cell types according to SLN status were also observed (Fig 2A and 2B, Table 3). Abundant inter-follicular and sub-capsular macrophages were present while macrophages were less abundant in follicles in both types of SLN. Higher densities of macrophages were found in SLN^+^ (Fig 2C and 2D, Table 3) which were characterized by high densities of sub-capsular macrophages compared to the SLN^-^. SLN^+^ invaded by numerous tumor cells, may contain melanophages (macrophages that have phagocyted melanosomes), as depicted in Fig 2A.

The densities of endothelial cells were high in SLN and increased in SLN^+^ (Fig 2). We found that 17/20 (85%) SLN^+^ were heavily stained (+++) with anti-CD34, while 22/41 (53%) of SLN^-^ display intense (+++) staining (Fig 2C and 2D).

### Positive and negative SLN from melanoma patients contain similar numbers of NK cells

**Fig 3:**
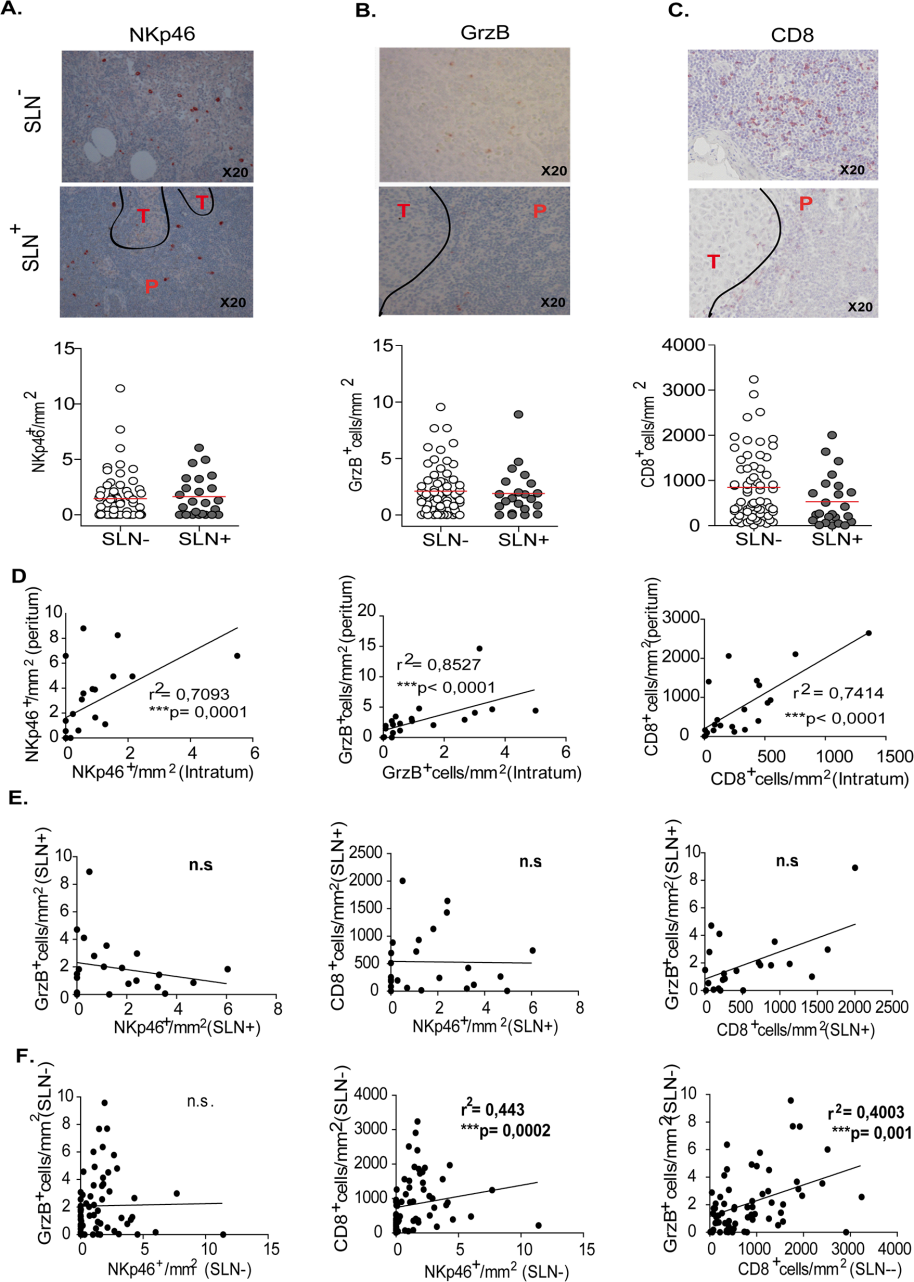
Immunohistological staining and enumeration of cytotoxic cells infiltrating SLN from melanoma patients. Representative staining and counts of immune cells in SLN^-^ and SLN^+^: (A) NKp46^+^ NK cells, (B) GrzB^+^ cells, (C) CD8^+^ T cells. (D) Correlation analyses of NKp46^+^ cells, GrzB cells and CD8^+^ T cell numbers between intra- and peritumor area in SLN^+^. (E, F) Correlation analyses between numbers of cytotoxic immune cells (NKp46^+^ cells, GrzB^+^ cells and CD8^+^ T cells) in the SLN^+^ (E) and in the SLN^-^ (F).

The numbers of NKp46^+^ NK cells were manually counted in multiple high power fields and averaged (Fig 3A). The staining with anti-NKp46 was bright red, detected on the membrane and in the cytoplasm in large granular NK cells that were present in low numbers as compared to primary cutaneous melanoma. We found similar numbers of NK cells in SLN^-^ and SLN^+^ (Fig 3A). In SLN^+^, NK cells were distributed in the peritumoral zones and rarely infiltrated the tumor clusters (Fig 3A).

The intensity of the staining of the SLN slides with the anti-GrzB mAb appeared light red (Fig 3B). The counting of GrzB^+^ cells indicated slightly higher numbers than NKp46^+^ cells in SLN and comparable numbers of GrzB^+^ cells were detected in SLN^-^ and SLN^+^ (Fig 3B). In SLN^+^, GrzB^+^ cells were more frequent in peritumoral than in intra-tumor areas (data not shown). In contrast, numerous CD8^+^ T cells were detected in SLN (Fig 3C). The staining was mainly on the membrane of CD8^+^ T cells that were scattered in the inter-follicular regions of the LN. Compared to SLN^-^, there was a trend for higher numbers of CD8^+^ T cells in SLN^-^ (Fig 3C). In SLN^+^, CD8^+^ T cells were more frequent in peritumoral areas and their numbers were lower in tumor cell clusters. A correlation was found between intratumoral and peritumoral NKp46^+^, GrzB^+^ and CD8^+^ T cell numbers (Fig 3D).

When SLN^-^ and SLN^+^ were analyzed independently, no correlation was found between the number of NKp46^+^, GrzB^+^ and CD8^+^ T cells in the SLN^+^ (Fig 3E). However, a correlation between NKp46^+^ and CD8^+^ T cells as well as between GrzB^+^ cells and CD8^+^ T cells was found in the SLN^-^ (Fig 3F). In addition, when the SLN^-^ and SLN^+^ were pooled, there was no correlation between NKp46^+^ cells and GrzB^+^ cells but the positive correlations between NKp46^+^ and CD8^+^ cells and between GrzB^+^ cells and CD8^+^ T cells were maintained, knowing that SLN^-^ numbers exceed the SLN^-^ ones (S2 Fig).

**Fig 4:**
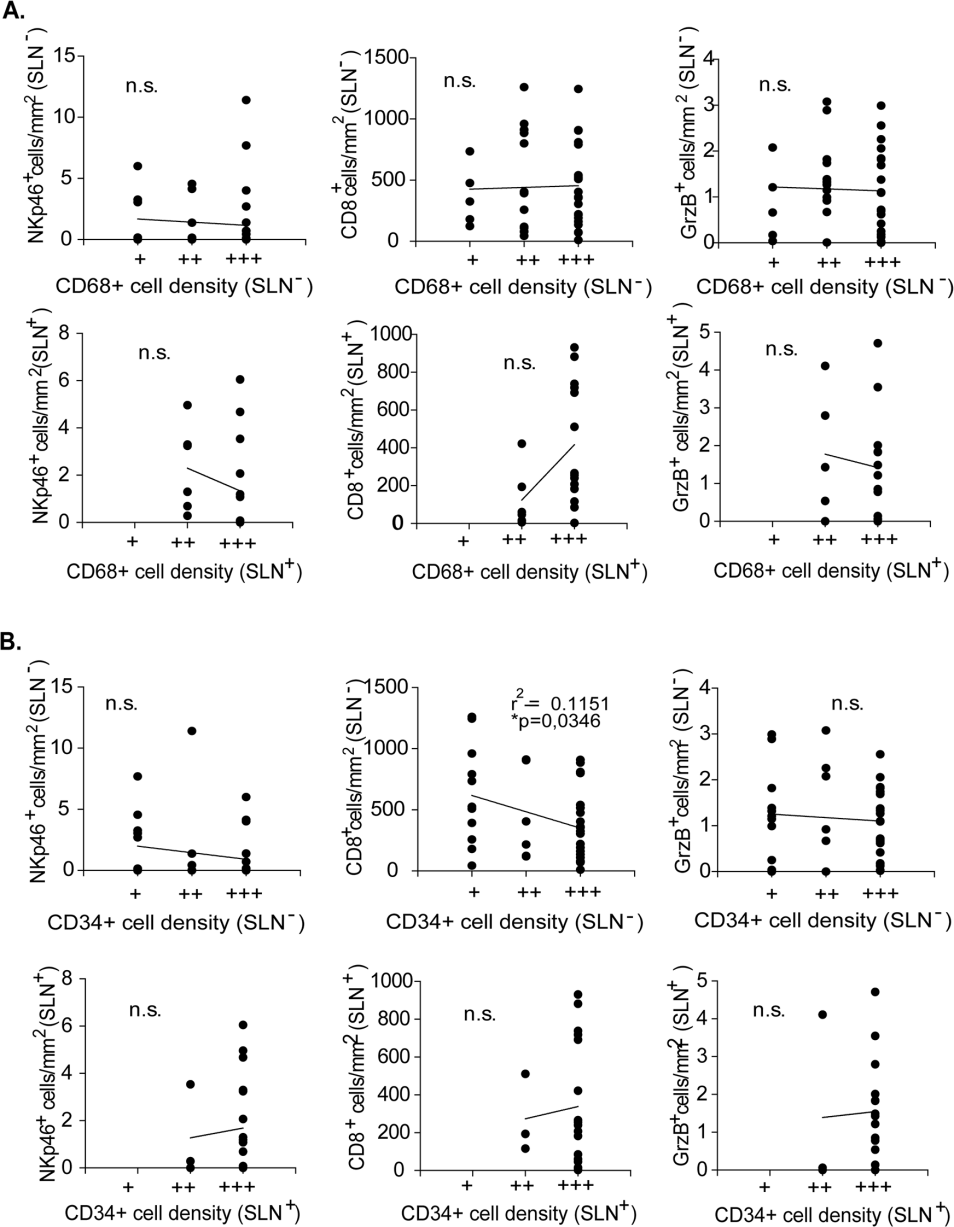
Correlation analyses between numbers of cytotoxic immune cells (GrzB^+^, NKp46^+^, CD8^+^ T cells) and the densities of (A) macrophages or (B) endothelial cells in SLN^-^ and SLN^+^.

Finally, relations between macrophages and endothelial cell densities and numbers of each immune cytotoxic cell subsets were analyzed in SLN^-^ and SLN^+^. No particular association between macrophages and cytotoxic cells was found (Fig 4A). We observed a negative correlation between CD8^+^ T cell numbers and the CD34^+^ cell densities in SLN^-^ not in SLN^+^ (Fig 4B).

### Infiltration of SLN by NK cells is associated to higher 5-years-relapses

The melanoma patients included in the present studies had a follow up period >5 years allowing correlation analyses with clinical evolution. In the SLN series, we found higher percentages of tumor relapses in SLN^+^ (48%) than in SLN^-^ (29%) group (Table 2).

**Fig 5:**
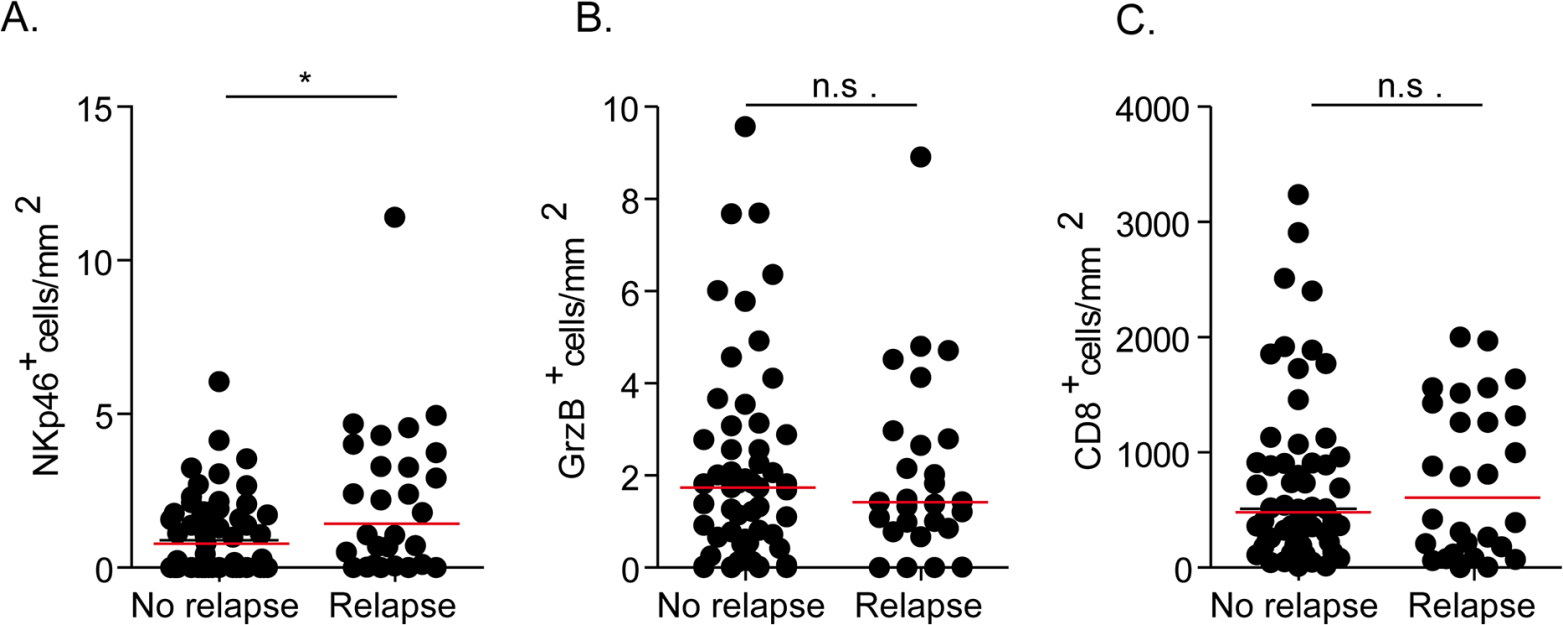
Association between numbers of cytotoxic (A) NK cells, (B) GrzB^+^ cells, CD8^+^ T cells (C) and 5-year-relapse rate of the patients.

Numbers of NKp46^+^ cells, Grz B^+^ cells and CD8^+^T cells showed no impact on the OS and the PFS (S3 Fig). However, while no correlation was found between the numbers of GrzB^+^ and CD8^+^T cells infiltrating SLN and the rate of 5 year-relapse, patients that had a relapse after the SLN procedure display a higher number of NKp46^+^ cells infiltrating SLN (Fig 5).

## Discussion

There are several recent studies indicating that NK cells infiltrate human solid tumors and that NK cell numbers have a prognostic value for patient evolution and/or response to treatment [14]. Positive prognostic value of tumor infiltrating NK cells has been reported in gastrointestinal tumors. Gastric cancer patients with high numbers of tumor infiltrating NK cells had tumor with lower depth of invasion, fewer metastases to LN and less lymphatic invasion and displayed higher rates of 5-year survival rate than patients with low levels of NK cells [15]. In gastric tumors, NK cells may be activated without DC or intratumoral lymphocytes. Intratumoral NK cells may act as independent immunologic effector against tumor cells [16]. Cytokine secreting CD56^bright^ NK cells accumulated in tumor foci of gastrointestinal stromal tumors (GIST) after Imatinib treatment and densities of NKp46^+^ cell infiltrates independently predicted PFS. Activated CD3^+^ Tumor infiltrating T Lymphocytes (TIL) in GIST were especially enriched in tumor maintaining class I MHC expression and predicted PFS in multivariate analyses, indicating that NK and T lymphocytes likely contributed independently to GIST immuno-surveillance [14].

In the present studies, we found that high numbers of NK cells infiltrate cutaneous melanoma and that NK cell numbers were not correlated with Breslow thickness, neither with clinical evolution but correlated with the age of the patients. It has been reported that older patients display cutaneous melanoma with more aggressive features but lower incidence of SLN metastases [17]. In the same series of tumors, stronger IL-27 staining was found in advanced thick melanomas and correlated with PDL-1 and IL-10 expression [18]. The data suggest that thick cutaneous melanomas display immunosuppressive features that may hamper NK cell activation and recruitment. Alternatively, host factors, in addition to age, may influence the presence of NK cells in cutaneous melanoma. Gene variability of Killer Ig like receptors (KIR) may also influence the disease susceptibility and prognosis of cutaneous malignant melanoma [19,20].

Recently, we have identified in metastatic LN a new subset of mature CD56^bright^CD16^+^ NK cells that display cytotoxic activities against melanoma cells following IL-2 or IL-15 stimulation [11]. To characterize the microenvironment of SLN draining cutaneous melanoma, we have investigated the densities of macrophages and CD34^+^ endothelial cells and counted cytotoxic NK cells, GrzB^+^ cells, and CD8^+^ T cells. Results were evaluated with regard to association with patient evolution and tumor parameters.

The present studies represent to our knowledge the first investigation of the distribution of NK cells in SLN of melanoma patients, using a specific mAb targeting NK activating receptor NKp46. In previous studies NK cells were detected with anti-CD57 or anti-CD56 mAbs that may also be expressed by activated T cells or non hematopoietic cells. We show that low numbers of NKp46^+^ NK cells infiltrate SLN corroborating our previous report on *ex vivo* analyses of macro-metastatic LN [11]. Furthermore, we found similar numbers of NK cells in SLN^-^ and SLN^+^, indicating that these cytotoxic cells do not accumulate when melanoma cells invaded the SLN.

Compared to NKp46, anti-GrzB mAb stained higher numbers of cells, and both NKp46^+^ cells and GrzB^+^ cells are distributed in the medulla surrounding the B follicles of the SLN. The absence of correlation between NKp46^+^ and GrzB^+^ cells suggests that at least certain NK cells infiltrating SLN may not be activated. Anti-GrzB mAb may label activated CD56^bright^CD16^+^perforin^+^ NK cells identified *ex vivo* in M-LN [11]. Anti-GrzB also stains small subsets of T cells.

The trend for higher numbers of CD8^+^ T cells and the correlation of CD8^+^ T and GrzB^+^ cell numbers in SLN^-^ suggest that an immune reaction is initiated in the LN. However, the rare detection of the cytotoxic cells in tumor cell clusters of SLN^+^ further suggests that a switch to immunosuppressive microenvironment as tumor grows may counteract this reaction. Previous studies indicated that TIL grade was an independent predictor of SLN positivity and survival [21–23]. The densities of TIL were estimated on HES slides, thus precluding distinction between cytotoxic and regulatory immune cells. Functional status of immune cells is important and transcriptional profiling of SLN identified exhausted CD30^+^ T cells associated with progression in melanoma patients [24].

High densities of macrophages and CD34^+^ cells in SLN, even more prominent in SLN^+^, may represent a favorable environment for metastatic invasion of SLN. An increased lymph fiow occurs before tumor cell invasion and was reported in SLN before melanoma cell invasion [25]. Macrophages were abundant in the sub-capsular and interfollicular areas and were detected inside tumor clusters of SLN^+^ exhibiting large melanoma invasion. In pancreatic tumors, M2-polarized tumor associated macrophages (TAM) density in regional LN was strongly associated with the nodal lymphatic vessel density, indicating that macrophages favor nodal lymphangiogenesis and promote LN metastasis [26]. SLN from breast cancer (BC) patients showed an accumulation of CD163^+^ in sinuses correlated negatively with CD8^+^ T cells in SLN^+^ [5]. We also observed high densities of endothelial cells and decreased CD8^+^ T cells numbers in SLN^+^ from melanoma. While CD68^+^ macrophages may favor melanoma cells invasion, DC accumulation and activation of CTL were present in SLN^+^ of melanoma patients with no other positive correlation in the downstream LNs [27].

Patients with documented clinical follow-up had allowed correlation analyses of the clinical evolution with the immunocyte infiltration of the SLN or the primary tumor. A recent meta-analysis indicated that primary tumor depth influences the impact of SLN status on OS and represent a better prognostic factor than SLN status [28]. In individuals with thin melanoma (<1 mm), SLN^-^ status conferred no survival advantage, while for intermediate depths, most studies reported worse survival in SLN^+^ melanoma patients, although the difference was not statistically significant. For thick melanoma (>4 mm), SLN positivity was related to worse prognosis [28,29].

The immune infiltrates in SLN had no impact on OS or PFS (S3 Fig). However, the numbers of NKp46^+^ cells infiltrating SLN correlated with higher rate of 5-year-relapse of patients whereas numbers of GrzB^+^ and CD8^+^ T cells did not, suggesting that immunosuppressive NK cells may infiltrate SLN. Previous examination of NK cell infiltration in LN draining gastric cancers, by staining with CD57 (expressed by activated NK cells and T cell subsets), showed comparable numbers of NK cells (as well as DC and CD3^+^ T cells) in SLN and non-SLN [30]. It is of note that numbers of CD57^+^ cells are close to numbers of GrzB^+^ cells we have detected in SLN, further indicating that anti-GrzB mAb may also detect some activated T cells. An additional study reported comparable infiltration by NK cells but accumulation of DC and activated T cells in negative LN from sub-mucosal cancer compared to invading gastric cancers. The authors conclude that decrease in immunocyte infiltration is associated with recurrence and that NK cells may be associated with anti-tumor effects in LN close to tumor [31].

In conclusion, few NK and GrzB^+^ cells infiltrate SLN (tumor free or with micro-metastases) draining primary cutaneous melanoma. Tumor invasion and high density of CD68^+^ and CD34^+^ cells increased in SLN^+^ may reflect a tumor favorable microenvironment.

## Supporting Information

S1 Fig (A) Kaplan-Meier curves were designed to analyze the association between the Breslow of the primary cutaneous melanomas of the SLN series patients and the overall survival (OS) and the progression free survival (PFS) of those patients. Correlation analyses between Breslow and the age of the patients (B), the ulceration of the primary cutaneous melanoma (C) and the 5 years-relapse (D). (E) Kaplan-Meier curves were designed to analyze the association between the SLN status and the OS and the PFS. (PDF)

S2 Fig Correlation analysis between the numbers of immune cytotoxic cells (NKp46^+^ cells, GrzB^+^ cells and CD8^+^ T cells) infiltrating the SLNs. (PDF)

S3 Fig Kaplan-Meier curves were designed to analyze theimpact of (A) the numbers of NK cells, (B) GrzB^+^ cells and CD8^+^ T cells (C) in the SLN series on the OS and the PFS. (PDF)
