# Supplementary figures and images for: Characterization of the Microenvironment in Positive and Negative Sentinel Lymph Nodes from Melanoma Patients
Source: PLoS One. 2015 Jul 28;10(7):e0133363. doi: 10.1371/journal.pone.0133363 (PMC4517810; doi:10.1371/journal.pone.0133363)

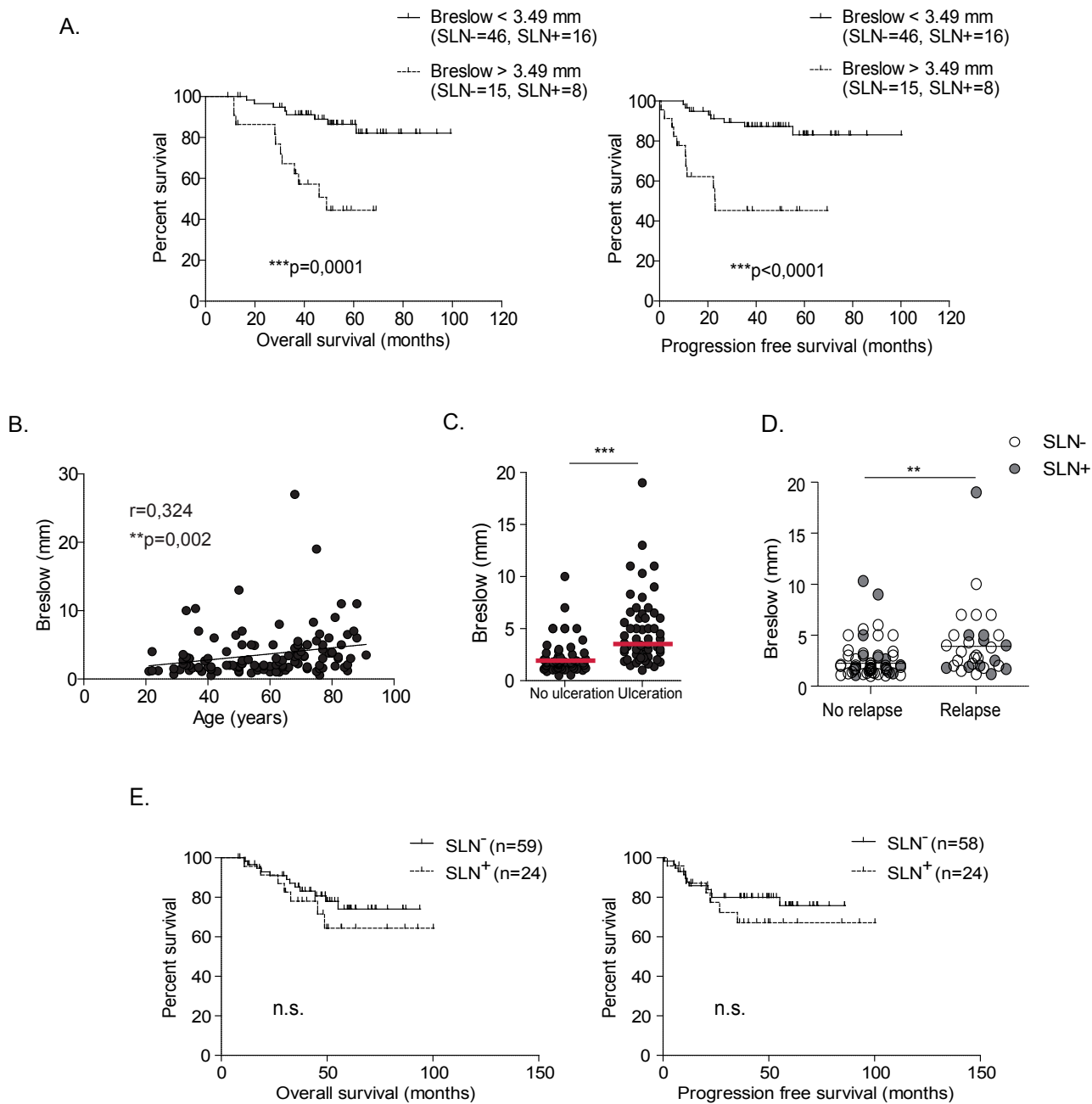

**Figure S1**

Supplement: S1 Fig — (PDF) [file pone.0133363.s001.pdf]

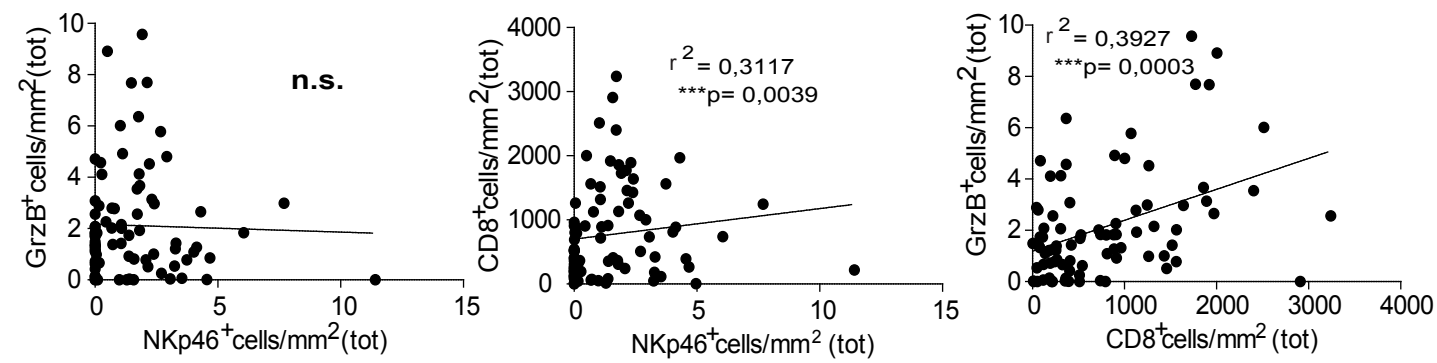

**Figure S2**

Supplement: S2 Fig — (PDF) [file pone.0133363.s002.pdf]

A.

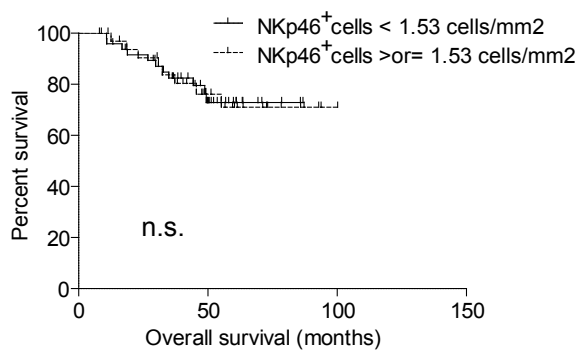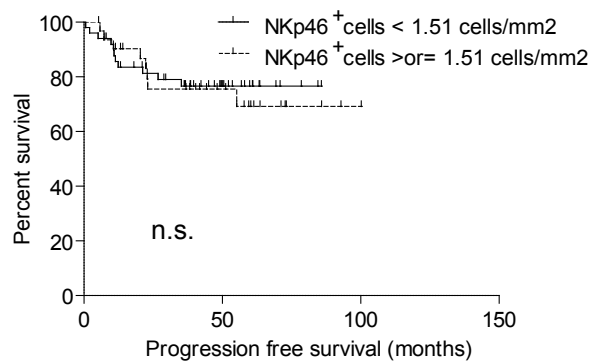

B.

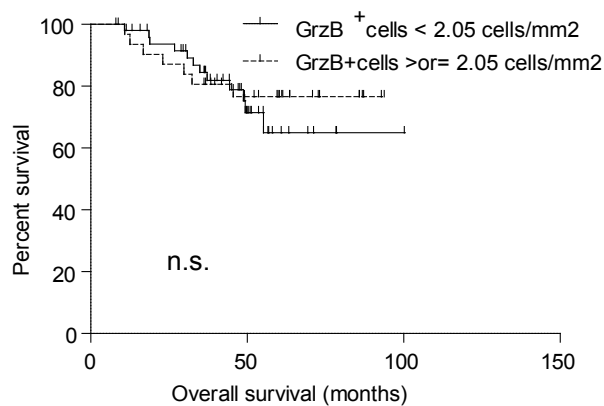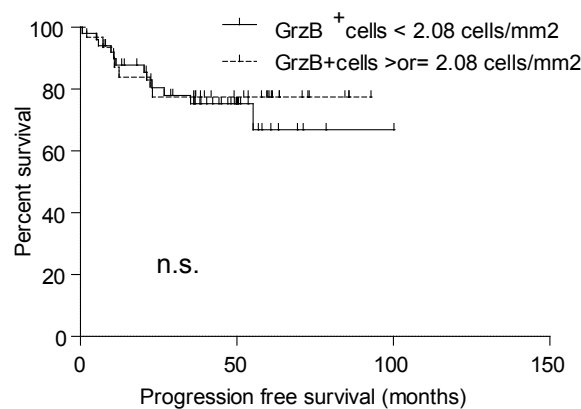

C.

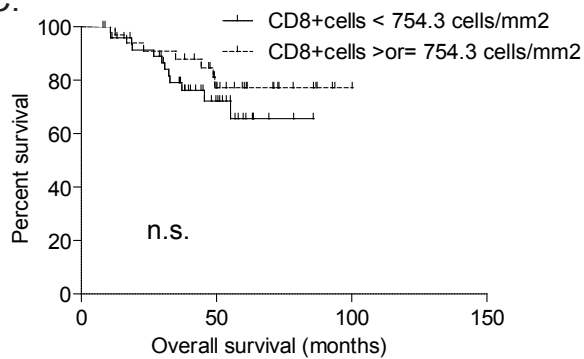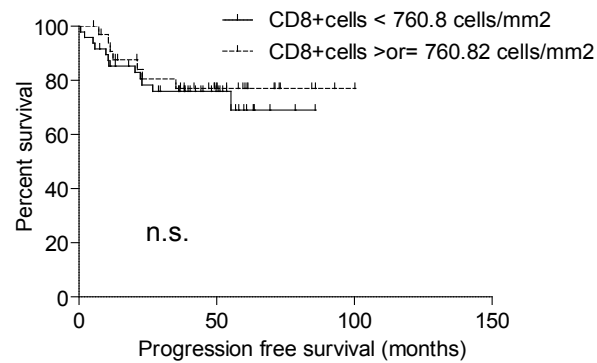

**Figure S3**

Supplement: S3 Fig — (PDF) [file pone.0133363.s003.pdf]
